# An evaluation of alertness training for older adults

**DOI:** 10.3389/fnagi.2014.00067

**Published:** 2014-04-15

**Authors:** Agnieszka Milewski-Lopez, Eleonora Greco, Flip van den Berg, Laura P. McAvinue, Sarah McGuire, Ian H. Robertson

**Affiliations:** ^1^Institute of Neuroscience, Trinity College DublinDublin, Ireland; ^2^TRIL Centre, University College DublinDublin, Ireland; ^3^Department of Psychology, University of LimerickLimerick, Ireland

**Keywords:** cognitive aging, alertness, sustained attention, metacognitive strategy, Self Alert Technique

## Abstract

We present an evaluation of a self-administered, biofeedback-aided, alertness training programme called the *Alertness: Training for Focused Living (ATFL) Programme*, which was developed as part of the Technology Research for Independent Living (TRIL) collaboration. We conducted two studies in order to evaluate the programme. A randomized controlled trial was, first of all, conducted with 40 older adults aged between 60 and 83. A series of five single case studies was then conducted to examine the suitability of the programme for use with people with more severe memory difficulties. In the randomized controlled trial, participants were assigned to the *ATFL* Programme or to a placebo programme. Aspects of participants' memory, attention and executive functioning were assessed via telephone prior to and following completion of the training programmes and at 1, 3, and 6-month follow-up sessions. Significant improvements in sustained attention and verbal fluency were noted in the *ATFL* group. The series of single case studies illustrated the importance of tailoring a programme to the needs and abilities of the clients in question. The potential benefits of the *ATFL* programme in terms of periodically boosting alertness and aiding executive functioning are discussed.

## Introduction

Normal aging is associated with decline in cognitive processes such as memory and executive functioning (West, 1996; Park, 2000; Park et al., 2001). Sustained attention, the ability to maintain alertness and focus over time (Sturm, 1996), is a cognitive process, whose intact functioning is fundamental to the functioning of more complex forms of attention, such as selective or divided attention (Sturm et al., 1997) and other forms of cognition, such as memory and reasoning (Craik, 2006). Essential to the maintenance of goal-directed behavior (Robertson, 2003; Robertson and Garavan, 2004), the ability to sustain attention under conditions of routine and low external demand has been found to be related to absentmindedness or everyday slips of memory and attention (Robertson et al., 1997; Smilek et al., 2010) and this capacity is measurable by a test, the Sustained Attention to Response Test (SART) (Robertson et al., 1997), which has been shown to be closely associated with the brain's noradrenaline system (Bellgrove et al., 2006; Greene et al., 2009).

In comparison to younger adults in their 20 and 30 s, adults in their 60 and 70 s have been found to make significantly more errors on the Sustained Attention to Response Task (McAvinue et al., 2012a) and older adults' performance on this task has been found to be significantly correlated with ratings of frailty (O'Halloran et al., 2014) and tendency to fall (O'Halloran et al., 2011). The current paper presents an evaluation of a training programme designed to provide older adults with a strategy to increase their alertness levels at will, in order to help them sustain attention to goal-directed activity.

The alerting network is one of the attentional networks posited in Posner and Petersens's (1990) tripartite model of attentional functions. Its function has been described in terms of achieving and maintaining an alert state, or a state of high sensitivity to incoming stimuli (Posner, 2008). The alerting network has been linked to a predominantly right hemisphere fronto-parietal network, which interacts closely with a subcortical arousal system (Robertson and Garavan, 2004; Raz and Buhle, 2006). At a cognitive level, the alerting network can be further parsed into phasic alertness, referring to the ability to increase response readiness for a short period of time subsequent to an external cue or stimulus, tonic or intrinsic alertness referring to a state of general response readiness (Sturm and Willmes, 2001) and sustained attention, referring to the endogenous maintenance of response readiness or task-relevant processing during monotonous tasks (Robertson et al., 1997).

The training programme evaluated here—*Alertness: Training for Focused Living (ATFL)*—was developed as part of the Technology Research for Independent Living (TRIL) programme (www.trilcentre.org). It aimed to teach older people how to recognize when their alertness levels were low in everyday life, and how to implement a specific strategy for temporarily increasing alertness in order to achieve key everyday life goals which they had identified. This was not a typical “brain training” intervention, but rather a strategy-based, metacognitively-oriented method for managing limited cognitive resources with specific reference to alertness and sustained attention.

The strategy that is at the core of the *ATFL* programme, Self Alerting, was originally devised for people suffering from unilateral neglect following right hemisphere stroke. Robertson et al. (1995) enhanced patients' ability to sustain attention to task by providing them with a mechanism to boost alertness periodically. Patients were first alerted with external sounds and gradually learned to produce the same effect in themselves using a self-instructional procedure. Robertson et al. (1995) found that the implementation of this strategy during various tasks led to improvements in unilateral neglect and sustained attention. The technique was further developed by O'Connell et al. (2008), who added biofeedback of skin conductance, a marker of autonomic arousal (Dawson et al., 2000), in order to aid participants' attempts to gain control of the alerting process. O'Connell et al. (2008) reported significantly improved sustained attention in adults with and without ADHD while Self Alerting during the Sustained Attention to Response Task. The Self Alert Technique introduced in the *ATFL* programme represents a further development of the technique, incorporating a shift in body posture and deep breaths in order to stimulate the autonomic arousal system (see McAvinue et al., 2012b).

The *ATFL* programme was designed to be a completely self-administered, 4 week programme consisting of a manual/guidebook, compact disc instructions and a specially designed skin conductance biofeedback device. This was sent to participants through the post and collected from them at the end of 4 weeks. As participants were cognitively assessed before and after training by telephone, there was no face-to-face contact between the research team and participants. A distinct feature of the *ATFL* programme was its careful design using ethnographic methodologies to ensure acceptability, usability and compliance by older adults.

We report the results of two studies, a randomized controlled trial designed to examine the efficacy of the *ATFL* programme in improving aspects of memory, attention, and executive functioning in a group of older adults and a series of single case studies designed to explore the suitability of the programme in addressing the needs of adults with subjective memory complaints and Mild Cognitive Impairment (MCI).

## Methods

In Study 1 (randomized controlled trial), forty older adults were randomly assigned either to the *ATFL* Programme, or to a placebo intervention, called the Attention Education Programme, both of which were self-administered training programmes lasting 4 weeks. Aspects of participants' memory and attention were tested prior to and after training and during three follow-up assessment sessions. In Study 2, a series of single case studies was conducted to examine the suitability of the programme for use with people with more severe memory difficulties. Five participants who were attending a memory clinic undertook the *ATFL* training programme and participated in pre-training, post-training, and follow-up assessment sessions. The same (or similar) measures of memory and attention were used but testing was conducted in person rather than over the phone. Participants and their partners (where appropriate) were interviewed and entries in the workbooks examined to ascertain participants' opinions on the programme, the Self Alert Technique and its utility or efficacy in everyday life.

### Participants

#### Study 1

Forty participants aged 60–83 (*F* = 21; *M* = 19; mean age = 72, *SD* = 6) were recruited through an advertisement in a local newspaper and through the TRIL clinic in St James's Hospital, Dublin. Participants were invited to participate if they had self-reported difficulties with everyday memory and attention, as evidenced by a combined score of more than 50 on the Revised Memory Failures Scale and on the Revised Attention-Related Cognitive Errors Scale (Carriere et al., 2008). Participants were only included if they were English Speaking, had self-reported good health, were without any neurological or psychiatric illnesses, and were living at home independently. Participants were excluded if they had Reynaud's disease, or any other circulation/movement condition affecting arms and hands, due to the results of a pilot study which showed how the presence of such conditions could disrupt a participant's experience of using the biofeedback device. This study was granted ethical approval by the relevant hospital and university ethical committees. Participants gave their informed consent prior to participation.

#### Study 2

Table 1 presents details for each participant. The first two participants had been diagnosed with MCI and their Mini-Mental State Examination (Folstein et al., 2010) scores are in keeping with this diagnosis. The remaining three participants had not been diagnosed with MCI but were attending the memory clinic with subjective memory complaints. Participant 3 complained in particular of word-finding difficulties.

**Table 1:** **Participant characteristics in Study 2**.

**ID**	**Sex**	**Age**	**MMSE**	**NARTIQ**	**Education**	**Memory complaint**
1	M	73	20	111	Secondary	Mild cognitive impairment
2	M	69	21	118	Secondary	Mild cognitive impairment
3	F	66	30	118	Undergraduate	Word-finding Difficulty
4	M	67	30	121	Postgraduate	Subjective memory complaints
5	M	68	29	123	Undergraduate	Subjective memory complaints

### Procedure

In Study 1 (randomized controlled trial), participants were randomly assigned to the Trainee or Control group using a computer algorithm minimization procedure which ensured the groups were matched for age and gender (Altman and Bland, 2005). Participants in the trainee group completed the *ATFL* Programme while participants in the control group completed the Attention Education Programme (see below). Participants were sent a copy of the appropriate programme by post. Cognitive assessment was conducted over the telephone, with participants being tested prior to engaging in the training, immediately after completing the programme and at three follow up sessions (1, 3, and 6 months after the end of the programme). Tests of memory and attention were chosen on the basis of ease of administration over the phone and their sensitivity to attention and memory impairments. All assessments were conducted over the phone by a trained research assistant, who was blind to group allocation. Participants were instructed to sit comfortably during the telephone assessment, to turn off mobile phones and to refrain from writing down anything that could assist their recall during the assessment. Scale cards were sent to participants in advance of testing to provide a visual support for the answer scales employed by the subjective measures. In Study 2 (single case series), all participants completed the *ATFL* Programme. A copy of the programme was given to each participant by the research assistant, who provided an overview of the programme in person. Assessment sessions (pre-training, post-training and follow-up) were conducted in person rather than over the phone.

### Materials

#### Training programmes

Both the *ATFL* and the Attention Education programmes were self-administered programmes, involving the use of a highly structured guidebook, which provided daily exercises, prescribed for 5 days of each week, for a period of 4 weeks. Both programmes were designed in a similar fashion, with each session involving a mixture of information to be read and questions to be answered or exercises to be performed. Both programmes informed participants about alertness and attention, the crucial difference between the two being that the *ATFL* Programme provided training in the use of Self Alerting.

***ATFL programme.*** A distinct feature of the *ATFL* programme was the rigorous process to which it was subjected while under development. Professionals with expertise in different disciplines, such as neuropsychology, interaction design and ethnography, collaborated in order to create a user-friendly technology solution. Their objective was to incorporate neuropsychological knowledge about alertness and the functioning of the brain while also designing a programme that reflected the needs and preferences of older adults. The latter was achieved through the use of an iterative participatory design process in which older adults were enlisted as co-creators of the programme. The *ATFL* programme passed through several pilot phases during which detailed feedback was sought from older adults who agreed to participate in the pilot programmes. Each round of feedback enabled further improvements of programme delivery protocol, guidebook content and presentation of the biofeedback interface. This detailed developmental process inspired confidence that the final product maximized the acceptability of the programme to older adults, its usability and their motivation for completing the programme. A detailed account of the development of the programme can be found at the following web address: http://www.trilcentre.org/images/homepage/focus-on/co-creating_an_alertness_training_programme.pdf

The *ATFL* programme was based on four key elements:

1. *Building a greater awareness of the concept of alertness*: During the first week of the Alertness Training Programme the guidebook supported the participants in building a greater awareness of their current alertness levels. The physical and mental aspects of the experience of alertness were explained and explored and participants were asked to create their own concept of alertness.
2. *Self Alert technique*. The core component of the Alertness Training Programme, the Self Alert Technique, was presented in Week two. Participants were taught to increase their alertness levels by Self Alerting, which involved the following components:
  - Sit upright in an alert position
  - Breathe in for a count of 3 and hold
  - Breathe out for a count of 3
  - Say “Focus” or a chosen alertness word to yourself
  In teaching the Self Alert Technique, instructions in the guidebook were supplemented by auditory instructions on a Self-Alerting Tutorial CD.
3. *Biofeedback device*. During week three of the programme the participant's experience of Self Alerting was augmented through the use of a biofeedback device, which enabled the participant to view changes in his/her physiological arousal levels as he/she Self Alerted. The purpose of this section of the training programme was to provide the participant with further practice in Self Alerting, to engender a deeper understanding of the process and effects of Self Alerting and to provide the participant with evidence for the validity of the technique in terms of its influence on physiological arousal.
  The device itself was designed in the form of a 12-inch cushion with an embedded device for recording and displaying Galvanic Skin Response (GSR) in real-time. Participants rested the cushion upon their laps and slipped their fingers into two Velcro loops which contained embedded sensors for recording GSR. Once the device was switched on, the participant's GSR was represented on the display by a circle, which expanded in size with increases in GSR.
4. *Goal Diary*. The focus of the last week was on goal-setting. Participants were asked to set “alertness goals” or to choose daily tasks or activities in which they felt their functioning could be improved through Self Alerting. They were asked to apply the Self Alert Technique during these activities and to monitor their performance in a Daily Goal Diary.

***Attention education programme.*** This programme contained the following components.

1. *General aspects of attentional processes*. The first week of the Alertness Education Programme involved a general introduction to the world of attention. Participants were provided with information on the different types of attention and the consequences of deficits in each attentional function.
2. *Alertness and the body*. The second week of the guidebook introduced the participants to the physiological correlates of attention and alertness, illustrating how the mind, body, brain and nervous system are affected by alertness.
3. *Alertness and Performance*. In week 3, participants were introduced to the idea of an “attentional curve,” which aided in the explication of how high and low alertness levels and how anxiety and relaxation affect performance.
4. *Attention and daily living*. The fourth week focused on the influence of attention and alertness in daily living, illustrating the importance of alertness for creating a more “focused life.”

#### Assessment measures

In Study 1, the pre-training, post-training and 1-month follow up assessments, which took approximately 40 min to complete, included all of the following measures. A shorter version of the assessment, which excluded the four questionnaires, was used for the 3 and 6 month follow-ups in order to reduce the duration of the testing to 20 min. In Study 2, participants attended only one follow-up session. A small number of additional measures were employed (see below).

#### Subjective measures

***Attention-Related Cognitive Errors Scale (ARCES; Carriere et al., 2008).*** The ARCES was used as a self-report measure of attention slips and absentmindedness in everyday life. It consists of 12 statements, each of which describes a particular instance of an attentional slip. Participants rate the frequency with which they experience such slips of attention along a scale ranging from 1 (never) to 5 (very often). Possible scores range from 12 to 60, with higher scores representing a higher degree of absentmindedness in everyday life.

***Memory Failures Scale (MFS; Carriere et al., 2008).*** Fashioned in the same way as the ARCES, the MFS is a self report measure of minor memory failures which occur in everyday life. Participants rate the frequency with which they experience memory failures described in a series of 12 statements, which are rated along a scale ranging from 1 (never) to 5 (very often). Possible scores range from 12 to 60, with higher scores representing a higher occurrence of memory failures in everyday life.

***The Toronto Hospital Alertness Test (THAT; Shapiro et al., 2006).*** The THAT is a 10-item self-report index designed to measure perceived alertness during the past week. Participants rate a number of items (e.g., the degree to which they felt “alert,” “fresh,” “energetic,” “able to think of new ideas”) along a 6-point scale, ranging from 0 (not at all) to 5 (all the time). Possible scores range from 0 to 50, with higher scores representing a higher degree of alertness.

***The Hospital Anxiety and Depression Scale (HADS; Zigmond and Snaith, 1983).*** The HADS was administered during each of the first three assessment sessions as a self-report measure of current psychological stress. It contains 14 items, half of which relate to feelings of anxiety and half, to feelings of depression. Participants rate the degree to which they were experiencing feelings expressed in each item during the past week, along a scale that is scored 0–3. Higher scores indicate a greater degree of psychological stress.

#### Objective measures

***Rivermead Behavioral Memory Test (RBMT; Wilson et al., 1985).*** Short passages of prose from the RBMT were used to assess immediate and delayed story recall. Different passages were used for each testing session. The research assistant read the passage aloud to the participant who was then asked to recall (verbally) as much as he/she could of the passage (immediate recall). Delayed story recall was assessed by asking participants to recall the passage once again 20 min later. Possible scores ranged from 0 to 21, with one point being given for each of 21 “ideas” recalled.

***Word list immediate and delayed recall.*** A pre-recorded list of 10 words was played to the participant at a constant rate of one word per second, three times, in the same order each time. After the third presentation, the participant was asked to recall as many of the words as he/she could. He/she was asked to recall the list once more after a delay of 20 min.

***Vigilant Auditory Attention Task (VAAT).*** The Sustained Attention to Response Task (SART; Robertson et al., 1997) is a test of visual sustained attention, which involves the presentation of numbers between 1 and 9 on computer screen. The participant's task is to press a button for every number that appears (Go-Targets) but to refrain from pressing for number 3 (the No-Go Target). In this auditory version of the SART, numbers are played by the computer over the phone and the participant is asked to say the word “TAP” upon presentation of each digit but to withhold the vocalization when he/she hears the number 3 being played. Using a fixed order of presentation (akin to SART_fixed_, McAvinue et al., 2005), 225 numbers were presented in sequential order (from 1 to 9), with an interval of 1.8 s from one digit to the next. Participants' vocalizations were recorded by the programme and errors of commission (saying TAP to a No-Go Target), errors of omission (neglecting to say TAP to a Go-Target) and reaction times, reflecting the onset of each vocalization, were recorded.

***Category Fluency (Cerhan et al., 2002).*** Category Fluency was used as a marker for executive functioning, tapping language and executive retrieval. Participants were asked to generate, within a 60-s time period, as many exemplars of specified semantic categories (animals, fruit and vegetables) as they could think of.

#### Additional measures in study 2

The following additional measures were used in Study 2.

***Mini-Mental State Examination −2 (Folstein et al., 2010).*** This is a brief 30 item questionnaire which taps areas such as orientation, memory, attention and language in order to screen for cognitive impairment.

***The National Adult Reading Test (NART; Nelson, 1982).*** An estimate of IQ was derived from performance on the NART, which comprises of 50 irregular words, which are read aloud and scored for accuracy.

***SART_Fixed_ (Robertson et al., 1997; McAvinue et al., 2005).*** This test of sustained attention was used instead of the VAAT, employed in Study 1. Numbers between 1 and 9 were presented in a fixed sequence on screen and participants were required to press a button for every number that appeared but to withhold their response to number 3. The numbers appeared in white against a black background. There were 225 digits in all, including 25 no-go targets (no. 3) and 200 go trials (all other numbers). The task lasted approximately 5.4 min. Two measures of sustained attention were calculated, namely errors of commission (i.e., number of times a participant pressed for no. 3) and errors of omission (i.e., number of times a participant did not press for a go-trial number).

## Results

### Study 1. randomized controlled trial

#### Objective measures

A mixed analysis of covariance (ANCOVA), including one between subjects variable, Group (two levels: trainee, control), one within subjects variable, Session (four levels: post-training assessment, 1-month follow-up, 3-month follow-up, 6-month follow-up) and one covariate, pre-training assessment, was run for each memory measure. Table 2 presents the mean values and standard deviations obtained by the trainee and control groups on each objective measure during each assessment session. It also presents the effect of Group across all post-training assessment sessions, obtained in the Mixed ANCOVA for each measure. In order to further examine the maintenance of training effects across follow-up sessions, *post-hoc* One-Way ANCOVAs were run to examine the effect of Group on each post-training assessment session separately. Table 3 presents the results in relation to the effect of Group for each of these analyses.

**Table 2:** **Means *(SDs)* for trainees and controls on objective measures from pre-training to 6-month follow-up, with main effect of group in mixed analysis of covariance (ANCOVA) examining effect of Group across all post-training assessments, including pre-training scores as covariate**.

		**Pre-training**	**Post-training**	**Follow up 1 month**	**Follow up 3 months**	**Follow up 6 months**	**Mixed ANCOVA: effect of group across all four post-training assessments**
Story recall immediate	Trainee	9.75 (3.23)	11.29 (2.81)	11.65 (3.16)	10.47 (3.57)	12.20 (2.80)	*F*_(1, 31)_ < 1, η^2^ = 0.03
	Control	9.88 (3.58)	12.28 (2.81)	14.13 (3.57)	10.68 (3.15)	11.29 (3.5)	
Story recall delayed	Trainee	8.80 (3.63)	10.00 (2.81)	9.85 (3.08)	8.88 (3.15)	10.90 (2.63)	*F*_(1, 31)_ = 1.53, *p* = 0.23, η^2^ = 0.05
	Control	8.78 (3.80)	10.95 (3.7)	12.83 (4.04)	9.53 (3.27)	10.42 (3.98)	
Word recall immediate	Trainee	8.85 (0.81)	8.47 (1.28)	8.35 (1.22)	8.62 (1.26)	8.53 (1.46)	*F*_(1, 31)_ = 3.6, *p* = 0.07, η^2^ = 0.1
	Control	8.60 (1.14)	9.00 (1.34)	9.15 (1.18)	8.79 (1.23)	9.16 (1.21)	
Word recall delayed	Trainee	6.50 (1.82)	7.06 (1.64)	6.41 (2.29)	8.12 (1.82)	8.00 (1.96)	*F*_(1, 31)_ < 1, η^2^ = 0.004
	Control	6.75 (1.94)	7.40 (1.96)	7.00 (2.00)	7.53 (2.29)	8.37 (1.77)	
VAAT ERC	Trainee	0.42 (1.22)	0.12 (0.33)	0.56 (0.73)	0.33 (0.62)	0.07 (0.27)	*F*_(1, 26)_ = 6.26, *p* = 0.02, η^2^ = 0.19
	Control	0.79 (1.18)	0.94 (1.31)	0.45 (0.51)	0.71 (0.69)	0.67 (0.77)	
VAAT ERO	Trainee	1.21 (2.96)	2.12 (6.88)	0.19 (0.75)	0.00 (0.00)	0.50 (1.61)	*F*_(1, 26)_ = 1.17, *p* = 0.29, η^2^ = 0.04
	Control	0.42 (0.61)	0.44 (0.86)	0.25 (0.44)	0.82 (1.81)	0.22 (0.55)	
VAAT RTcov	Trainee	0.15 (0.05)	0.13 (0.04)	0.13 (0.04)	0.13 (0.04)	0.13 (0.05)	*F*_(1, 26)_ = 1.16, *p* = 0.29, η^2^ = 0.04
	Control	0.15 (0.04)	0.14 (0.04)	0.13 (0.04)	0.15 (0.05)	0.14 (0.04)	
Category fluency	Trainee	13.45 (3.12)	14.82 (3.8)	18.12 (3.48)	13.63 (3.1)	12.93 (4.57)	*F*_(1, 31)_ = 1.43, *p* = 0.24, η^2^ = 0.04
	Control	13.80 (4.51)	11.80 (3.81)	16.30 (4.14)	13.53 (3.63)	14.89 (5.01)	

**Table 3:** ***Post-hoc* ANCOVAs examining effect of Group for each post-training assessment separately, including pre-training scores as covariate**.

	**Post-training assessment**	**Follow up 1 month**	**Follow up 3 months**	**Follow up 6 months**
Story recall immediate	*F*_(1, 34)_ = 1.09, *p* = 0.31,	*F*_(1, 34)_ = 5.68, *p* = 0.02,	*F*_(1, 32)_ < 1,	*F*_(1, 31)_ = 1.01, *p* = 0.32,
	η^2^ = 0.03	η^2^ = 0.14	η^2^ = 0.003	η^2^ = 0.03
Story recall delayed	*F*_(1, 34)_ < 1,	*F*_(1, 34)_ = 6.94, *p* = 0.01,	*F*_(1, 32)_ < 1,	*F*_(1, 31)_ < 1,
	η^2^ = 0.02	η^2^ = 0.17	η^2^ = 0.02	η^2^ = 0.006
Word recall immediate	*F*_(1, 34)_ = 2.04, *p* = 0.16,	*F*_(1, 34)_ = 5.27, *p* = 0.03,	*F*_(1, 32)_ < 1,	*F*_(1, 31)_ = 3.08, *p* = 0.09,
	η^2^ = 0.06	η^2^ = 0.13	η^2^ = 0.01	η^2^ = 0.09
Word recall delayed	*F*_(1, 34)_ < 1,	*F*_(1, 34)_ < 1,	*F*_(1, 32)_ = 2.66, *p* = 0.11,	*F*_(1, 31)_ < 1,
	η^2^ = 0.004	η^2^ = 0.01	η^2^ = 0.08	η^2^ < 0.001
VAAT ERC	*F*_(1, 32)_ = 5.81, *p* = 0.02,	*F*_(1, 32)_ < 1,	*F*_(1, 29)_ = 2.08, *p* = 0.16,	*F*_(1, 28)_ = 6.17, *p* = 0.02,
	η^2^ = 0.15	η^2^ = 0.01	η^2^ = 0.07	η^2^ = 0.18
VAAT ERO	*F*_(1, 32)_ < 1,	*F*_(1, 32)_ < 1,	*F*_(1, 29)_ = 2.98, *p* = 0.1,	*F*_(1, 28)_ < 1,
	η^2^ = 0.004	η^2^ = 0.001	η^2^ = 0.09	η^2^ = 0.03
VAAT RTcov	*F*_(1, 32)_ = 1.81, *p* = 0.19,	*F*_(1, 32)_ < 1,	*F*_(1, 29)_ = 1.2, *p* = 0.28,	*F*_(1, 28)_ < 1,
	η^2^ = 0.05	η^2^ = 0.02	η^2^ = 0.04	η^2^ = 0.02
Category fluency	*F*_(1, 34)_ = 12.94, *p* = 0.001,	*F*_(1, 34)_ = 2.79, *p* = 0.1,	*F*_(1, 32)_ < 1,	*F*_(1, 31)_ = 1.37, *p* = 0.25,
	η^2^ = 0.28	η^2^ = 0.08	η^2^ = 0.002	η^2^ = 0.04

**Figure 1:**
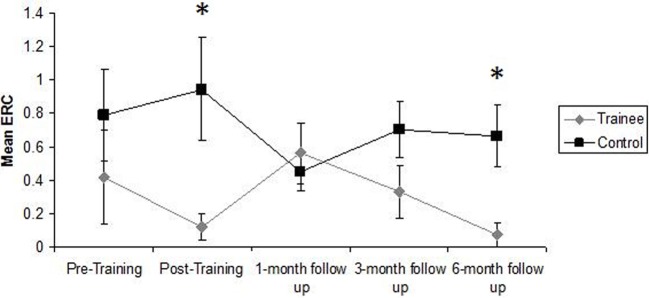
**Mean errors of commission committed by trainees and controls on the VAAT during each assessment session**. Error bars represent standard error of the mean. ^*^Statistically significant effect of Group (*p* < 0.05) in One-Way ANCOVA.

There was a statistically significant effect of training, with small effect size, η^2^ = 0.19, on VAAT ERCs, *F*_(1, 26)_ = 6.26, *p* = 0.02 (see Table 2). Trainees made significantly fewer errors than controls in the post-training assessment, *F*_(1, 32)_ = 5.81, *p* = 0.02, η^2^ = 0.15. While the difference between trainees and controls was not statistically significant during the 1-month, *F*_(1, 32)_ < 1, and 3-month follow-ups, *F*_(1, 29)_ = 2.08, *p* = 0.16, they were again found to make significantly fewer errors than controls during the 6-month follow-up assessment, *F*_(1, 28)_ = 6.17, *p* = 0.02, η^2^ = 0.18 (see Table 3). These findings are clearly illustrated in Figure 1.

**Figure 2:**
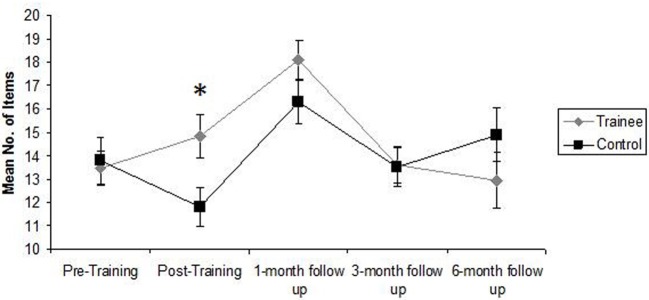
**Mean number of items recalled by Trainees and Controls during Category Fluency Tasks during each assessment session**. Error bars represent standard error of the mean. ^*^Statistically significant effect of Group (*p* < 0.05) in One-Way ANCOVA.

There was also a significant effect of training on Category Fluency. There was a statistically significant effect of Group during the post-training assessment session, *F*_(1, 34)_ = 12.94, *p* = 0.001, η^2^ = 0.28, with trainees recalling a significantly higher number of items than controls, relative to baseline scores (see Table 3). The effect of Group was not statistically significant across all post-training assessments, *F*_(1, 31)_ = 1.43, *p* = 0.24, however, indicating that this improvement was not maintained across follow-up sessions (see Table 2). These findings are clearly illustrated in Figure 2.

The effect of training was not statistically significant for Immediate or Delayed Story Recall, Immediate or Delayed Word Recall or EROs and RTCov on the VAAT.

#### Subjective measures

Similar analyses were performed for the subjective measures, the difference being that these were limited to only two follow-up sessions (as explained above). Table 4 presents the mean values and standard deviations obtained by the trainee and control groups on each subjective measure during each assessment session. It also presents the results in relation to the effect of Group for each of the ANCOVAs. It is clear from Table 4 that, with one exception, there was no significant difference between trainee and control groups during any post-training assessment session in terms of the self-reports. The exception was the HADS score during the 1-month follow-up, *F*_(1, 34)_ = 6.11, *p* = 0.02, η^2^ = 0.15, which indicates that relative to baseline, trainees reported a significantly higher level of psychological stress than controls during the 1-month follow-up.

**Table 4:** **Means *(SDs)* for trainees and controls on subjective measures from pre-training to 1-month follow-up and main effect of group in analyses of covariance (ANCOVA)**.

		**Descriptive statistics**			**Mixed ANCOVA**	**One-Way ANCOVA**	
		**Pre-training**	**Post-training**	**Follow up 1 month**	**Effect of group across both post-training assessments**	**Post-training**	**Follow up 1 month**
ARCES	Trainee	35.50 (3.76)	33.41 (3.74)	34.76 (3.33)	*F*_(1, 34)_< 1, η^2^ = 0.007	*F*_(1, 34)_ = 1.35, *p* = 0.25, η^2^ = 0.04	*F*_(1, 34)_< 1, η^2^ = 0.002
	Control	35.00 (7.37)	34.15 (7.32)	33.55 (8.04)			
MFS	Trainee	32.50 (5.05)	30.94 (4.72)	30.06 (4.34)	*F*_(1, 34)_< 1, η^2^ = 0.008	*F*_(1, 34)_< 1, η^2^ < 0.001	*F*_(1, 34)_ = 1.05, *p* = 0.31, η^2^ = 0.03
	Control	32.60 (6.15)	30.50 (6.21)	31.00 (6.34)			
THAT	Trainee	30.35 (6.56)	30.24 (5.07)	30.94 (4.26)	*F*_(1, 34)_< 1, η^2^ = 0.009	*F*_(1, 34)_< 1, η^2^ = 0.002	*F*_(1, 34)_< 1, η^2^ = 0.01
	Control	30.15 (4.74)	30.45 (4.43)	31.50 (4.02)			
HADS	Trainee	10.90 (4.53)	10.18 (3.34)	11.24 (4.22)	*F*_(1, 34)_ = 3.30, *p* = 0.08, η^2^ = 0.09	*F*_(1, 34)_< 1, η^2^ = 0.02	*F*_(1, 34)_ = 6.11, *p* = 0.02, η^2^ = 0.15
	Control	9.55 (4.72)	8.75 (4.33)	8.60 (3.72)			

*Main effect of group in mixed analysis of covariance (ANCOVA) examining effect of Group across both post-training assessments, with post-hoc One-Way ANCOVAs examining effect of Group for each post-training assessment separately*.

### Study 2. single case series

Table 5 presents participants' scores on each of the subjective and objective measures during the pre-training, post-training and follow-up assessment sessions. While firm conclusions about the effects of the *ATFL* programme on these measures cannot be drawn from an examination of individual performance, these scores clearly show the difference in cognitive function between those participants diagnosed with MCI (Participants 1 and 2) and those with subjective memory complaints (Participants 3–5).

**Table 5:** **Participants' scores on each test and questionnaire during pre-training, post-training and follow-up assessment sessions**.

**ID**	**Session**	**Word recall immediate**	**Word recall delayed**	**Story recall immediate**	**Story recall delayed**	**Fluency**	**SART ERC**	**SART ERO**	**ARCES**	**MFS**
1	Pre	5	0	5.5	0	3	5	20	43	50
	Post	5	1	7	3	7	7	15	44	36
	Follow	4	0	7	2	4	5	18	47	54
2	Pre	6	0	3	1	6	4	23	41	43
	Post	4	0	2	2	6	1	8	29	31
	Follow	8	3	3	1.5	4	11	7	43	41
3	Pre	10	8	8	7	14	1	2	36	26
	Post	10	9	7.5	7.5	19	2	0	29	23
	Follow	10	9	8	8	18	2	1	27	22
4	Pre	9	8	16.5	15	17	1	3	33	36
	Post	10	9	18	14.5	22	0	0	34	32
	Follow	10	9	16.5	14.5	13	3	2	38	34
5	Pre	10	10	14.5	13	8	2	2	30	24
	Post	10	10	14	13.5	15	1	0	31	30
	Follow	10	9	15.5	15.5	16	1	1	32	30

Table 6 presents a summary of each participant's experience of the *ATFL* programme and the Self Alert Technique, along with its impact on their daily lives. There was a clear divide between the participants in terms of their overall experience of the programme. The three participants who had subjective memory complaints but otherwise unimpaired cognitive function, all reported a generally positive experience of the programme. For the two participants with MCI, the experience of participating in the programme was described as very stressful by their wives. The root of the stress lay with the difficulty in completing the daily workbook exercises, which were simply too taxing for these participants.

**Table 6:** **Participants' experience of the *ATFL* Training Programme and Self Alert Technique**.

**ID**	**Experience of *ATFL* programme**	**Experience of self alert technique**	**Alertness goals**	**Goal achievement**	**Incorporation of self alerting into daily life**
1	Stressful	Increased awareness and clarity of thoughts Increased control of emotions and body Calming effect	Keeping track of conversation Expressing thoughts clearly Remembering people's names Concentrating on task at hand	Self alerting effective in helping to keep track of conversation and improving performance of specific tasks, e.g., weeding the garden	No
2	Stressful	Enjoyable Positive effect on thoughts and emotions Calming/Relaxing effect on body	Remembering people's names Keeping up with conversations Improving mental dictionary Getting things done/Improving concentration	Self alerting effective on a number of occasions, helping him to “get things done,” to remember a purchase previously made and to “feel less bothered”	Yes
3	Positive	A calming and relaxing effect on thoughts, emotions and body	Being able to recall something that she has just read or heard Being able to express her thoughts Keeping track of conversations	Tried with mixed success on several occasions, including listening to a talk, before reading the newspaper, during conversation and when feeling anxious and stressed	Occasional use for relaxation
4	Positive	Increased clarity and focus of thoughts Calmer and controlled emotions Energised body	Motivating self for action Solving daily living problems Getting things done Retrieving vocabulary to express thoughts clearly Remembering people's names	Positive effect on motivation for action	No
5	Positive	Created mental image of himself examining or doing something with complete absorption	Remembering people's names Starting a project Listening to others properly Focus exclusively on current tasks	Positive effect on motivation for action Somewhat effective in maintaining concentration	No

In terms of the experience of Self Alerting itself, in general, participants reported similar effects, describing an increase in clarity of thought and a calming, relaxing or controlling effect on the emotions or the body. Participants also set similar goals, the most common being remembering people's names, keeping track of conversations, concentrating on tasks, expressing thoughts clearly and enhancing motivation for action. All participants reported at least some degree of success in relation to achieving their goal activities by applying the technique in daily life. Despite this and the generally positive effects ascribed to Self Alerting, at follow-up only one participant had managed to incorporate the technique for regular use in daily life. Participant 1's wife explained that her husband's difficulties in completing the programme had reduced his willingness to implement the technique in daily life. In contrast, Participant 2, despite experiencing similar difficulties completing the programme, had managed to incorporate the technique. He had written the simple steps of Self Alerting on a small piece of paper, which he kept in his wallet and used several times a day as a prompt and reminder of the steps involved. He reported that engaging in Self Alerting, with the aid of this written prompt, had had a beneficial effect on his daily cognitive functioning.

Participants 3–5 all expressed an intention to continue to use Self Alerting in their workbooks. At follow-up, however, they all reported that they had largely failed to do so, partly due to a unanimous conclusion that they did not really need the technique. Participant 3 described occasionally Self Alerting in order to relax. For example, she found it to be effective in offsetting her anxiety at times when she was struggling to find words. She described how it gave her the time to focus and “get the words out.” Participant 4 blamed a lack of self discipline for his failure to incorporate the technique and Participant 5 admitted that he was not always sure when to employ the technique and mentioned that it was not always possible or appropriate to find the few minutes needed to engage in Self Alerting during daily activities or situations.

## Discussion

In this paper, we have presented an evaluation of a training programme—*Alertness: Training for Focused Living (ATFL)*—designed to train participants in the use of the Self Alert Technique, a strategy which can be used to periodically boost alertness at will in order to sustain attention during goal-oriented activity. We presented the results of a randomized controlled trial in which the effects of the programme on aspects of older adults' memory, attention and executive functioning were compared to the effects of a control programme. In a second study, we conducted a series of single case studies to explore the utility and efficacy of the *ATFL* training programme and the Self Alert Technique for use in daily life by participants with more severe memory complaints.

The results of the randomized controlled trial indicated that participation in the programme led to a statistically significant increase in trainees' performance on the VAAT and in category fluency, in comparison to control participants. This enhanced performance in sustained attention and executive functioning was generally restricted to post-training assessments and was not maintained at follow-up (with the exception of a reappearance of a significant difference between trainees and controls on the VAAT during the 6-month follow-up assessment). There were no significant effects of participation in the training programme on episodic memory (for words or prose) or self-reported alertness or slips of memory and attention.

The improvements in sustained attention and executive functioning are actually quite notable given that the objective of the *ATFL* programme was not to train the cognitive processes of alertness or sustained attention *per se*, but to provide participants with a strategy to boost alertness at will. Participants were not prompted to Self Alert during the post-training assessment sessions and their improvements must therefore represent a general effect of participation in the programme, which possibly led to a generally enhanced level of alertness, rather than implementation of the Self Alert Technique itself during assessment. The lack of maintenance of the improvements makes sense in this context. It is also possible that effects on memory would be discernible if participants were prompted to employ the Self Alert Technique throughout the assessment session. This is for a future study to investigate.

The *ATFL* programme could potentially benefit older adults in two ways. First, it provides a technique to enhance alertness in order to sustain attention in the service of goal-directed action. As such, it joins Goal Management Training (Robertson, 1996) as one of the few strategies that are available to aid executive functioning in older adults. First developed within the context of brain injury, Goal Management Training was designed to aid executive functioning by encouraging patients to “stop and think” about task demands, prompting them to define the task before them, identify the subtasks involved in a complex task and monitor their performance. Levine et al. (2000) reported significant improvement in the performance of patients with Traumatic Brain Injury on a set of real-life tasks following Goal Management Training. More recently, Levine et al. (2007) reported on the efficacy of a combination of Goal Management, memory and psychosocial training in improving the performance of healthy older adults on simulated real-life tasks. It has been suggested that older adults rely more upon executive functions in order to compensate for declining sensory and perceptual processing (Madden and Whiting, 2004; Madden et al., 2005; Madden, 2007; McAvinue et al., 2012a). Strategies aimed at supporting executive functioning are particularly important in this context. The *ATFL* programme could easily be combined with strategic training, such as Goal Management, providing a mechanism for enhancement of alertness prior to engaging in the problem-solving process.

The second way in which the *ATFL* programme may be of benefit to older adults is through the provision of repetitive boosts of noradrenaline. The underlying biological mechanisms of Self Alerting, evidenced by the documented increase in skin conductance (O'Connell et al., 2008), is a surge of noradrenaline which mediates an enhancement of autonomic arousal. It has been suggested that noradrenaline, through a variety of neuroprotective mechanisms, such as neurogenesis and synaptogenesis, may be the biological mechanism that mediates cognitive reserve, a term used to describe the protection from brain pathology afforded by variables such as education, intelligence and mental stimulation (Robertson, 2013). Along with the current study, a number of studies have illustrated that the alertness system is highly amenable to stimulation through both endogenous and exogenous means (Robertson et al., 1995, 1998; Sturm et al., 1997, 2004, 2006; Thimm et al., 2006, 2009; O'Connell et al., 2008; DeGutis and Van Vleet, 2010; Hauke and Sturm, 2011; Finke et al., 2012). It could be that, regardless of task application, frequent boosts of alertness, through Self Alerting or other means, would have a beneficial effect on the brain function of older adults.

The results from the series of single case studies illustrate the importance of using a participatory design process in order to tailor a training programme to the needs and abilities of the clients in question. The *ATFL* programme was designed with the collaboration of healthy older adults and the results of the single case studies suggest that participation in the current programme was simply too taxing for the two participants with MCI. This may have been due to the content of the programme, which was designed to be challenging and motivating for participants, including, for example, explanations of complex concepts such as the role of noradrenaline in alertness. Indeed, during the pilot phases, the learning element emerged as one of the main motivators for older adults to continue with the programme. This level of cognitive content may have overwhelmed the participants with MCI.

Despite difficulties experienced while working through the guidebook, the second participant had managed to incorporate Self Alerting into his daily life. He had done so with the aid of a written prompt detailing the steps of Self Alerting, which he kept in his wallet. This finding suggests that a much simplified programme, focussing solely on the mechanics of the Self Alert Technique and practice in using it in daily life may be beneficial for participants with MCI. Given that these participants can lack awareness of their difficulties, it may also be beneficial to involve their carers to a greater extent, by creating a mutually empowering programme which may maintain motivation and persistence better than a self-directed programme. Such development would require entry into a new participatory design process with participants with MCI and their carers.

All three participants with subjective memory complaints expressed an intention to incorporate Self Alerting into their daily lives but at follow-up, none had managed to do so. Reasons given for this included a feeling of not needing the technique, a lack of self discipline and uncertainty regarding when and how to employ the technique. Participants with fewer memory complaints may benefit from an extended period of applying the technique to their goals. The current programme allots only 1 week for this activity and this may not be enough to enable participants to see the benefit of Self Alerting and to fully incorporate the technique into their lives.

Overall, the current pair of studies supports the potential of the *ATFL* programme as a method for enhancing the alertness of older adults. The *ATFL* programme could be further developed by creating a much simpler version for participants with MCI, with an explicit involvement of their carers, and by extending the training period during which participants apply the technique during their goal activities. A future study could better assess the impact of the technique on memory, attention and executive functioning by incorporating prompts for Self Alerting during the post-training assessment sessions. An effort to assess ecological validity, by asking participants to keep diaries of everyday attention, memory or action slips before, during and after training, would also be warranted.

## Funding

This research was completed as part of a wider programme of research within the TRIL centre (Technology Research for Independent Living). The TRIL Centre is a multi-disciplinary research centre, bringing together researchers from University College Dublin, Trinity College Dublin, NUI Galway and Intel, funded by Intel, IDA Ireland and GE Healthcare. See www.trilcentre.org.

### Conflict of interest statement

The authors declare that the research was conducted in the absence of any commercial or financial relationships that could be construed as a potential conflict of interest.
